# Measuring Health Inequalities Using the Robin Hood Index: A Systematic Review with Meta-Analysis

**DOI:** 10.3390/epidemiologia6030035

**Published:** 2025-07-10

**Authors:** Georgios Farantos, Athanasios Pitis, Maria Diamantopoulou, Fotini Tzavella

**Affiliations:** Department of Nursing, School of Health Sciences, University of the Peloponnese, 22100 Tripoli, Greece; a.pitis@go.uop.gr (A.P.); m.diamantopoulou@go.uop.gr (M.D.); tzavella@uop.gr (F.T.)

**Keywords:** health inequalities, general practitioners, indexes, resource allocation

## Abstract

**Background/Objectives:** Although the Robin Hood Index (RHI) is increasingly used to quantify geographic health inequality and guide resource redistribution, empirical evidence on whether higher physician density reduces RHI-measured inequality remains limited. This study systematically reviews and meta-analyzes RHI-based research to assess the association between physician distribution and health inequalities. **Methods:** We conducted a systematic review and meta-analysis of studies using the RHI to evaluate health inequalities, without restrictions on country or publication date. Following PRISMA 2020 guidelines and registered in PROSPERO (CRD42024496486), we searched PubMed, Scopus, and OpenGrey literature, extracted data on physician density and RHI outcomes, and conducted a meta-analysis. Odds ratios (ORs), ln(OR), and 95% confidence intervals (CIs) were calculated, and risk of bias was assessed using the Robvis tool. **Results:** Seventeen studies covering 720 regions and 1.07 billion individuals were included. Three clusters emerged: physician redistribution (10 studies), poverty–mortality associations (six studies), and systematic reviews (one study). Physician redistribution was strongly associated with increased inequality and policy attention (r = 0.73; *p* = 0.0038). Meta-analysis of eight redistribution studies yielded a pooled OR of 1.24 (95% CI: 0.54–2.86), consistent in sensitivity analysis (OR = 1.26; 95% CI: 0.56–2.89). Poverty–mortality studies also showed a correlation with the number of variables considered (r = 0.59; *p* = 0.022). **Conclusions:** A greater physician supply is associated with increased health inequalities, with statistical support but limited certainty. Methodological heterogeneity in RHI-based studies constrains comparability. Standardized methodologies and broader analytic models are needed to inform research and guide health policy.

## 1. Introduction

Measuring health inequalities with the Robin Hood Index (RHI) is a modern alternative method (De Maio, 2007 [1]; Kawachi & Kennedy, 1997 [2]) calculated based on the Lorenz curve (Mobaraki et al. [3]; Kennedy et al. [4]) and is used to reallocate resources (Wilkinson & Symon, 2000 [5]; Honarmand et al., 2017 [6]). Measuring health inequalities with RHI is also used in combination with other methods, such as the Gini index (Kennedy et al. [4]) or even Data Envelopment Analysis (Dlouhý, 2018 [7]).

A framework for measuring health inequalities was developed by De Maio (2007) [1] and Lynch et al. (2004) [8], who argue that the literature provides limited support for confirming the direct effect of income inequality on health. Inequality regarding people’s access to resources is measured by calculating geographical inequalities, in many cases using the Robin Hood Index (RHI) or the Pietra ratio. The RHI is based on the Lorenz curve and shows the resources that need to be transferred from rich individuals to poor individuals to achieve equality, as described by Kennedy et al. (1996) [4]. Although these studies explore inequalities using RHI, they approach the issue from different perspectives, which suggests the need for further investigation to provide a more comprehensive understanding of how RHI can be applied across diverse healthcare systems and regions.

The Robin Hood Index is a measure of health inequalities that is equivalent to the maximum vertical distance between the Lorenz curve and the line of equality (Theodorakis et al., 2006, [9]). Health inequalities, as evidenced by Rezaei et al. (2015) [10], are a global concern. Health inequality is related to the proper or improper distribution of physicians in different geographical areas of the same territory (Honarmand et al., 2017) [6]. The density of physicians and health workers, in general, determines the health service delivery outcomes to the population (Mobaraki et al., 2013) [3].

In both developed and developing countries, a phenomenon exists where physicians cluster in metropolitan centers (Honarmand et al., 2017) [6]. In most developing countries, the proportion of physicians and nurses in rural areas is limited to 23% and 38%, respectively (Mobaraki et al., 2013) [3]. The free market allocates health resources according to the ability to pay, rather than according to population health needs, resulting in underserved areas in poorer regions (Dlouhý, 2018) [7].

Gravelle et al. [11] highlight one aspect of inequality: agencies measure inequality using medically underserved areas, where the population is steadily declining, which appears to reduce inequality. Dlouhý [12] emphasizes the importance of policy changes and accompanying regulatory measures in achieving the redistribution of physicians to achieve equity in health services. The equity criterion is pursued by some governments in policy-making, even as the cost of healthcare continues to rise (Farantos & Koutsoukis, 2023) [13].

According to (Kawachi & Kennedy, 1997) [2], the mechanisms by which income inequalities that extend to health service provision are associated with worse health status are identified in:

- Underinvestment in human capital;
- Loss of social cohesion;
- Disinvestment in social capital;
- Potentially harmful consequences of the frustration caused by such deprivation.

Szwarcwald [14] found significant correlations between income distribution and health indicators.

There are studies on the effect of health inequalities measured by RHI, as well as a much older systematic review on this topic, which does not directly answer our question. The aims of this article are to systematically review and meta-analyze studies that measure inequalities using the RHI method.

Despite the growing body of research on health inequalities, there remains a significant gap in studies that specifically focus on the application of the Robin Hood Index (RHI) across different regions and healthcare systems. While various studies have explored the relationship between income distribution and health outcomes (Kawachi & Kennedy, 1997 [2]; Kennedy et al., 1996 [4]; Lynch et al., 2004 [8]), few have systematically analyzed the impact of health service distribution using RHI as a primary measure particularly in relation to the effect of unequal distribution of physicians across different regions. Additionally, the existing literature predominantly addresses health inequality from a theoretical standpoint (De Maio, 2007 [1]; Honarmand et al., 2017 [6]; Lynch et al., 2004 [8]), with limited empirical evidence on the effectiveness of policy interventions aimed at redistributing healthcare resources among healthcare professionals. This highlights the need for a more comprehensive and systematic review of studies that utilize the RHI method, particularly in the context of resource allocation in underserved areas. Such an analysis could provide valuable insights into how health inequalities manifest in both developed and developing countries and guide future policy decisions.

Beyond studies that employ the Robin Hood Index (RHI) or other formal measures of health inequalities, several investigations have reached similar conclusions through alternative methodological approaches. Specifically, the literature that does not utilize explicit inequality indices has also identified a relationship between changes in the number of physicians and variations in health inequalities. Inadequate planning of the medical workforce can lead to a reduction in the number of physicians and, consequently, to an increase in health inequalities. The Lithuanian experience demonstrates that changes in the number and specialization of physicians—such as the significant decline in key specialties—can impact indicators related to healthcare disparities (Šablinskas & Stankūnas, 2024) [15]. The expansion of medical schools in Brazil has led to an increase in the number of physicians per 1000 inhabitants, particularly in underserved and more remote municipalities, contributing to a reduction in regional healthcare access inequalities. This targeted growth in the physician workforce has strengthened health infrastructure and improved the equitable distribution of healthcare professionals (Figueiredo et al., 2019) [16]. The implementation of the More Doctors Program led to an increase in the number of physicians in underserved and vulnerable municipalities, thereby enhancing the quality of primary healthcare services. This increase in physician availability contributed to reducing healthcare inequalities by promoting greater equity and expanding healthcare coverage in disadvantaged areas (Silveira et al., 2020) [17]. The existing literature, including studies without explicit inequality indices, consistently demonstrates that changes in the number and distribution of physicians significantly influence health inequalities, with workforce reductions exacerbating disparities and targeted expansions improving equity in healthcare access and quality.

Our systematic review addresses the research gap created by the heterogeneity observed in previous studies and the lack of a comprehensive analysis of health inequalities related to the distribution of physicians across regions using the Robin Hood Index (RHI). While some studies explore aspects of physician distribution, no synthesis has yet provided a unified view of how RHI can be applied in this context. By integrating and analyzing the available evidence, our work will fill this gap, contributing to the broader evidence ecosystem and offering insights to inform future research and policy on healthcare disparities.

Research question: Does increasing the number of physicians lead to a reduction in health inequalities, as indicated by studies that calculate inequalities using the RHI?

Secondary research question: Do RHI studies in hospitals reach homogeneous conclusions about health inequalities?

## 2. Materials and Methods

This study is a systematic review and meta-analysis of studies that measure health inequalities using the Robin Hood Index (RHI). We followed the PRISMA 2020 guidelines for systematic reviews and the JBI evidence synthesis manual for the meta-analysis process. This research was registered in the PROSPERO database (PROSPERO 2024, number CRD42024496486) to ensure transparency and adherence to predefined protocols for systematic reviews. Studies were selected based on inclusion criteria that focused on the assessment of health inequalities using the Robin Hood Index (RHI), including systematic reviews and meta-analyses. However, studies were excluded if they used the RHI in combination with other indicators or if they did not specifically focus on health inequalities.

The detailed database search strategy, including specific keywords, inclusion and exclusion criteria, and search terms used, is provided as a Supplementary File S1. The Supplementary File S1 provides a comprehensive description of the search strategy used in this systematic review and meta-analysis. It outlines the process of identifying both published and unpublished studies on measuring health inequalities with the Robin Hood Index (RHI). A key focus of the file is the detailed presentation of the search strategies for the PubMed and Scopus databases, as well as the gray literature strategy for OpenGrey. The file includes search strategy tables for both PubMed and Scopus, which display the search queries, the number of records retrieved, and the applied date and language limitations. Additionally, it highlights the data extraction tool, presented as a table, which organizes relevant concepts and methodological variables for analyzing the selected studies on health inequalities.

We only included published studies, published in English (since none were found in French).

Outcomes: To be included, a paper had to reference the measurement of health inequalities using the RHI. Any methodology relevant to the topic under study is acceptable.

We have scored the data on an appropriate scale to facilitate its processing.

We used the PRISMA 2020 checklist to verify the items included in this analysis.

The meta-analysis was conducted in accordance with Chapter 1 of the *JBI Evidence Synthesis Manual* for the category “JBI Systematic Reviews” [18].

The extracted data was systematically classified into several categories, including the use of the RHI, the relationship between physician redistribution and health outcomes, and the methodological variables applied in each study. These categories included indicators such as the use or non-use of the RBI and the Gini index. Regarding the object in relation to targeting, studies were categorized based on whether they focused on the redistribution of physicians or examined the relationship between poverty and mortality. Additionally, we identified studies that explicitly linked their findings to policy targets. In terms of methodological variables, we distinguished between different types of variables, ranging from health outcomes to adjustments for physicians. The data was also classified according to the unit of analysis, with some studies focusing on communities, others on individuals, and some extending over time.

The results from the studies were grouped into key outcomes, including the increase in the number of physicians, the connection between health and poverty, and the general redistribution of healthcare workers according to the RHI. Other important categories included the reference RBI for areas with a low number of doctors, the RHI for areas with a high number of physicians, and the RHI growth rate.

Finally, the conclusions of the studies were categorized based on findings related to the distorted distribution of physicians, the significance of the RHI, and its correlation with policies. Emphasis was also placed on issues such as downgraded divisions and inequality. Moreover, we extracted data related to the search terms used in the studies, particularly with respect to the use of RBI, references to health equality and inequality, and the application of analytical statistics with *p*-values.

To evaluate the risk of bias, we used the Robvis-I V2 risk of bias tool [19], and where relevant, trial protocols were reviewed for discrepancies between the published results and study protocols. To assess the risk of bias due to incomplete results in a synthesis, we proceeded to compare results between the included papers. We studied the trial protocols when they were available in published form. Any information that was included in the trial protocols was compared with the information in the final publication. Whenever required, we requested the test protocols. We performed a final assessment of the risk of bias of incomplete results. The final assessment of the risk of bias was performed after comparing all available data.

We used the methodology of Dlouhý [7] and Lynch, Smith, Harper, Hillemeier, Ross, Kaplan, Wolfson [8] to classify the study findings in our study. This classification helps to draw conclusions from our systematic review.

A conversion of the data was carried out. Some data were converted into numerical codes to allow for their processing.

**Figure 1:**
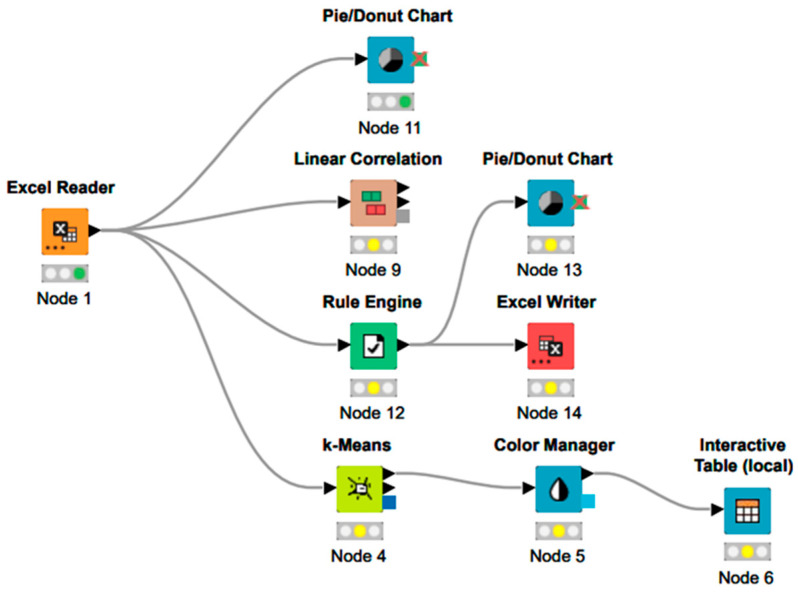
Workflow in KNIME used for data extraction and meta-analysis.

The data analysis was conducted using KNIME software (version 4.7.1), where we performed clustering and generated graphical representations of the results. We constructed a workflow for this analysis. This workflow is shown in Figure 1. We performed clustering of similar data and produced graphical and statistical measures of the data analysis. Pearson’s correlation coefficient and *p*-value statistical significance coefficient were calculated using the KNIME software.

Additionally, the meta-analysis was conducted following the guidelines of the JBI Systematic Reviews, and heterogeneity was explored using forest plots generated from the software results. We conducted a study based on the work by [20] to estimate the effect of physician variation on changes in health inequalities. We calculated the meta-analysis measures (odds ratio, ln(OR), Upper 95% CI, and Lower 95% CI). We constructed the forest plot data and the forest plot required to estimate this variation. The investigated intervention is the increase in the number of physicians relative to a reference value. The change in health inequality values represents the outcome.

We used a random-effects model to account for expected heterogeneity among included studies, given the differences in study design, population, and variables analyzed. The summary odds ratio (OR) and 95% confidence intervals were computed using inverse-variance weighting. Meta-analysis and forest plot visualization were performed using Excel.

Heterogeneity was assessed using the I^2^ statistic, which quantifies the proportion of variation across studies due to heterogeneity rather than chance. Values of I^2^ > 50% were interpreted as moderate to substantial heterogeneity. Given the small number of included studies (*n* = 8), formal subgroup or sensitivity analyses were not feasible.

When synthesizing the results, heterogeneity was examined through the study of the forest plot and the results from the KNIME software, allowing the studies to be categorized. We employed a method to investigate potential causes of heterogeneity among study results.

We studied the methodologies of [21,22], applying the KNIME to health inequalities.

To assess the certainty in the evidence set for an outcome, we examined the homogeneity of the evidence and methods used in each outcome. In addition, we examined the software used in the studies.

We hereby confirm that generative artificial intelligence (GenAI) tools were not used in the preparation of this manuscript. All aspects of the research—including study design and implementation, preliminary and main literature searches, data processing methods, and analytical strategy—were conducted entirely using traditional approaches. Literature research was performed manually using validated electronic databases on a standard computer workstation, and no AI-generated text, data, or graphics were employed at any stage.

## 3. Results

### 3.1. Correlation of Inequalities with Disease Indicators

**Figure 2:**
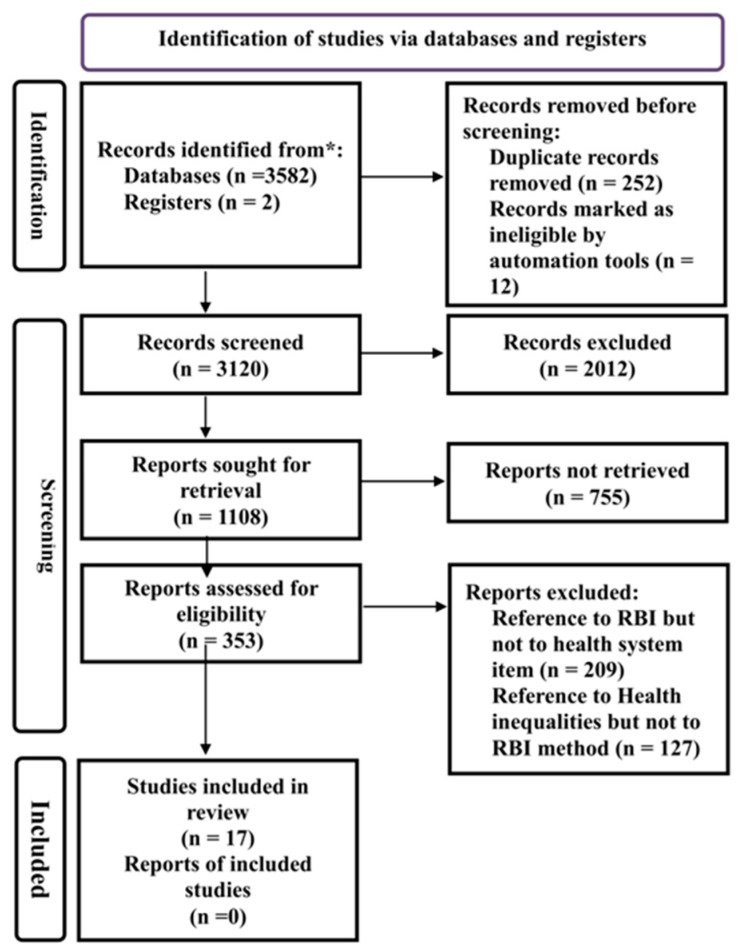
PRISMA 2020 flow diagram of study selection process inequalities [6,9,10]. * We separated studies from databases and registries to distinguish published and registered records.

Seventeen published studies were reviewed, encompassing 720 geographical regions and a total of 1,073,904,189 individuals (the sum of the total population across all studies). Figure 2 shows the Prisma flow diagram for our study.

We identified several studies that focused on the association between inequalities and disease indicators [4,23], and several studies, to some extent, challenge the above results [2,14]. The redistribution of physicians, especially GPs, to a greater or lesser extent, seems to be a common component that most of the reviewed studies conclude.

Some studies unambiguously demonstrate an increase in health inequity, even though the number of physicians increases over the same period [3,24]. Additionally, some studies are exploring the methods of estimating and interpreting inequality [7,11].

Some studies suggest that increasing the number of physicians can improve health.

### 3.2. Variables of the Reviewed Studies

Table 1 shows a summary of the research variables as derived from the literature review of the studies.

### 3.3. Clustering

According to the assessment carried out with the Robvis risk of bias tool, no extensive risk of bias was found for the examined studies, nor was there a risk of missing results.

From processing in the clustering branch, we extracted the clusters:

- Cluster 1 (redistribution of the number of physicians) includes 10 studies.
- Cluster 2 (systematic reviews) includes one study.
- Cluster 3 (poverty–mortality studies) includes six studies.

### 3.4. Correlations

Across clusters, a consistent pattern emerged: studies using more variables tended to report stronger statistical methods and greater reference to inequality outcomes. Some interesting correlations emerge: studies using RBI and Gini simultaneously show a high correlation with reporting on health policies (r = 0.727, *p*-value = 0.0038). In the group of poverty–mortality studies (*n* = 6), we examined whether the number of analytical variables considered per study (e.g., income, mortality, smoking, fertility) was associated with two key factors: (a) the use of formal statistical correlation methodologies (such as Pearson correlation or regression with *p*-value reporting), and (b) explicit references to health policy implications. Both were coded as binary variables (1 = presence, 0 = absence). A statistically significant positive correlation was found between the number of variables and the use of formal statistical correlation methods (r = 0.609, *p* = 0.016), as well as between the number of variables and policy relevance (r = 0.59, *p* = 0.022). These *p*-values reflect the significance of Pearson correlation coefficients computed among the six studies in this cluster, indicating that studies with richer variable sets tend to also employ more rigorous statistical techniques and engage with policy discourse.

A higher number of analytical variables reported per study was found to be significantly associated with several key outcomes: negatively with the presence of distorted physician distribution (r = −0.471, *p* = 0.023), and positively with references to income redistribution (r = 0.616, *p* = 0.027) and the extent of RHI utilization (r = 0.56, *p* = 0.036). These patterns suggest that studies incorporating a greater number of analytical variables tend to employ more formal statistical methods and offer richer interpretations of inequality outcomes. These correlations suggest that studies incorporating a broader range of variables tend to report more detailed policy-relevant findings related to healthcare inequality.

Studies that find an increase in the number of physicians often report, in their results, a distorted distribution of doctors (r = 0.718, *p*-value = 0.044). Finally, studies using statistical methodologies using *p*-value show a high correlation with the health-poverty connection (r = 0.609, *p*-value = 0.016), the emphasis on income redistribution (r = 0.491, *p*-value = 0.018), the analysis of the importance of measuring inequalities using the RBI index (r = 0.777, *p*-value = 0.015) and using the RHI (r = 0.751, *p*-value = 0.0029). Such findings suggest that methodological depth may co-occur with greater policy sensitivity, though the small sample size of clusters limits generalizability.

**Figure 3:**
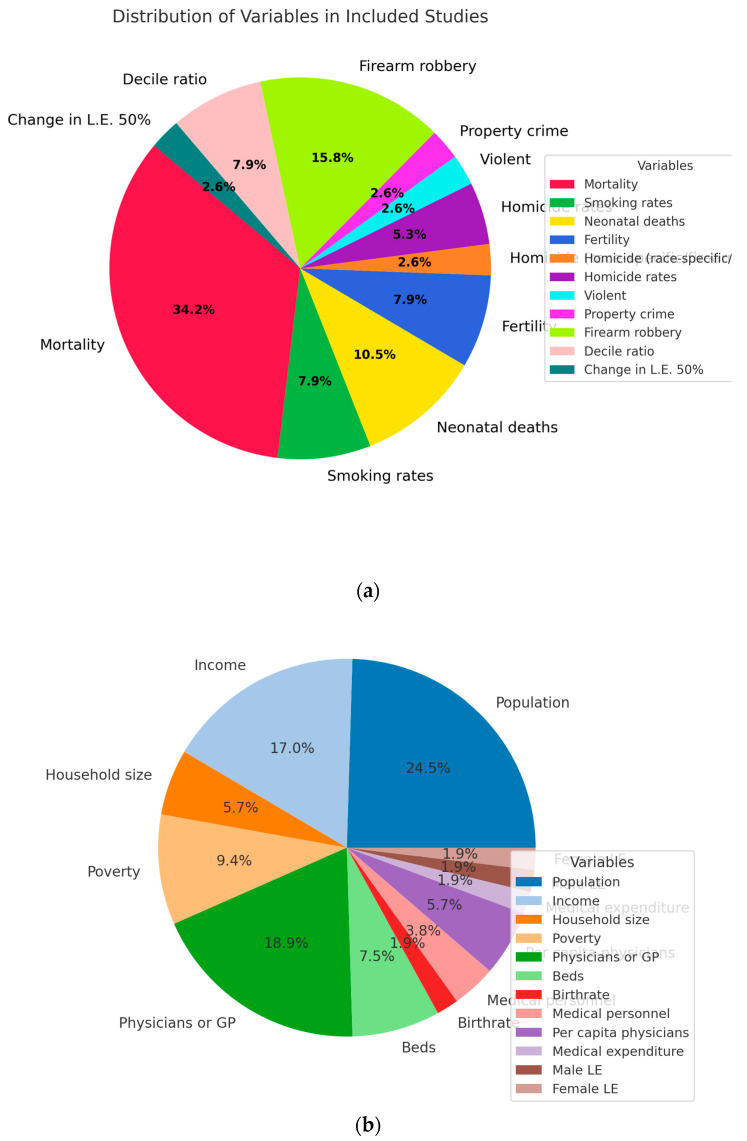
Distribution of variables in the included studies: (**a**) overall variables; (**b**) adjusted variables.

Figure 3 illustrates the distribution of both outcome and adjustment variables across the studies included in the review. As illustrated in Figure 3a, general mortality emerged as the most frequently reported health outcome across the included studies, followed by firearm-related indicators (e.g., robbery, violence), fertility rates, neonatal mortality, and smoking prevalence. These variables reflect the heterogeneity of endpoints used to assess health inequalities in relation to the distribution of resources.

In terms of covariates used in statistical adjustment (Figure 3b), the most common were total population size, the number of physicians or general practitioners, income level indicators, and measures of poverty. These adjustment variables were extracted from the methodological frameworks of each study and used to control structural, demographic, or economic variation. The distribution shown reflects both the frequency of use and the conceptual emphasis placed on these variables by researchers applying the RHI to health equity analysis. These patterns reflect the methodological emphasis of the literature on demographic and structural determinants when evaluating health inequalities using the Robin Hood Index.

### 3.5. Meta-Analysis

Some of the studies included in our systematic review have relatively small sample sizes, which could potentially limit the generalizability and statistical power of the findings. For example, the study by Omrani-Khoo [26], with a sample size of only 18,585 individuals, and the study by Beck [27], which included 167,653 individuals, are considerably smaller compared to other studies that encompass millions of participants. These smaller studies may introduce bias or reduce the robustness of the overall conclusions. To mitigate the impact of these studies on the meta-analysis results, we excluded them from the meta-analysis process. This decision was made to ensure that the final outcomes accurately reflect more statistically robust studies with larger sample sizes, thereby improving the precision and reliability of the results presented.

**Figure 4:**
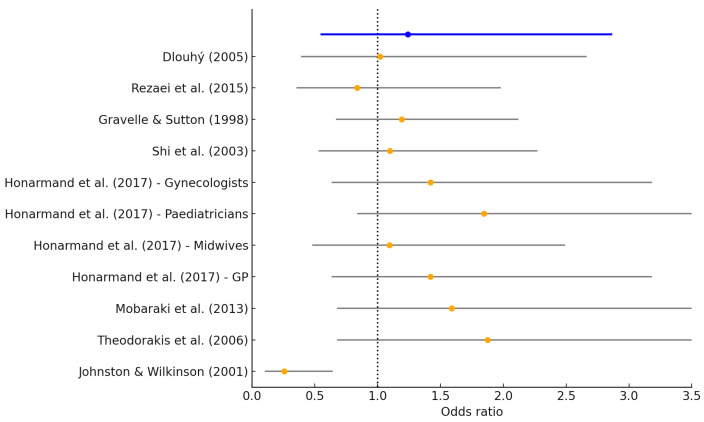
Association between the increase in the number of physicians and the rise in healthcare inequality across the reviewed studies. Νote: The blue line shows the odds ratios along with their natural logarithms and 95% confidence intervals (upper and lower limits) [3,6,9,10,11,12,24,25].

We calculated the odds ratios along with their natural logarithms and 95% confidence intervals (upper and lower limits) and plotted the relationship between the increase in physicians and the increase in inequality in the forest plot. The forest plot is shown in Figure 4.

While individual studies show variability, the pooled OR provides a broader perspective on the aggregate direction and magnitude of the association. The forest plot summarizes the results of eight studies that assessed the relationship between physician redistribution and health inequalities using the Robin Hood Index (RHI). The x-axis represents the odds ratio (OR), which quantifies the likelihood that an increase in physician density is associated with an increase in inequality. Values above 1 suggest a positive association (i.e., higher physician supply linked to greater inequality), while values below 1 indicate a potential reduction in inequality.

Figure 4 also presents individual OR estimates and their corresponding 95% confidence intervals (CIs). The studies demonstrate variation in direction and magnitude, with most indicating a trend toward increasing inequality. The combined summary effect is represented by the final diamond-shaped marker in the plot, yielding a pooled OR of 1.24 (95% CI: 0.54–2.86). This suggests a tendency toward increased inequality with physician growth, although the confidence interval includes the null value (OR = 1), and thus the result does not reach statistical certainty. Although the overall OR exceeds 1, indicating a potential link between physician redistribution and increased inequality, the wide confidence interval suggests caution in interpretation.

The overall pooled odds ratio (OR) derived from the meta-analysis is 1.24 (95% CI: 0.54–2.86). This OR reflects the likelihood that studies reporting increases in physician supply also report increases in health inequality, as measured by the Robin Hood Index. An OR greater than 1 suggests a positive association—in this context, it implies that greater redistribution of physicians may coincide with higher observed inequality rather than its reduction. This finding, although counterintuitive, may reflect the possibility that redistribution interventions are implemented in areas already experiencing entrenched disparities.

However, the confidence interval is wide and includes the null value (OR = 1), which indicates statistical uncertainty. This suggests that, although a trend toward increased inequality is observed, the available evidence does not allow us to conclusively affirm a causal or consistent effect. The heterogeneity across studies and the methodological variability in RHI measurement likely contribute to this uncertainty.

To enhance readability, OR values were rounded to two decimal places, and a vertical reference line at OR = 1 was added to indicate the threshold for no effect. The forest plot serves to visually synthesize heterogeneity across studies and to emphasize the need for further high-quality analyses with standardized adjustment methods. Of the physician redistribution studies, most (n = 8) show statistical significance in the positive effect of increasing the number of physicians on increasing inequality. However, we noticed that none of them are within the limits of certainty. One study demonstrated statistical significance in the association between an increase in physicians and a decrease in inequality. This study also falls short of statistical certainty. Together, the correlation and meta-analysis findings emphasize the importance of both study design and variable selection in shaping how the Robin Hood Index captures health inequalities. Heterogeneity was assessed using the I^2^ statistic. The adjusted I^2^ value was 67.2%, indicating moderate to substantial heterogeneity across studies. This supported our decision to use a random-effects model for synthesis.

### 3.6. Sensitivity Analysis

In the results of the meta-analysis, before the sensitivity analysis, the overall average odds ratio is 1.24020 (CI: 0.54–2.86). We conducted a sensitivity analysis by removing the study of [7], as it did not meet the criteria: it was authored by a single writer, not indexed in PubMed and Scopus databases, and used no-adjusted indicators. After this, the overall average odds ratio was adjusted to 1.26240 (CI: 0.56–2.89). A second sensitivity analysis was performed by further removing five studies that were checked against less stringent criteria for the use of adjusted indicators, resulting in an overall average odds ratio of 0.72306 (CI: 0.38–1.38).

## 4. Discussion

### 4.1. Summary and Interpretation of the Main Findings

We observed that studies measuring health inequalities with RHI use variables, many of which are repeated mortality [2,3,5,6,8,9,14,23,24,25], population [3,4,5,6,7,9,10,11,12,14,24,25,26,27], and the number of GPs [3,5,6,7,9,10,11,12,23,24,25,26]. This finding is important for the selection of variables in future research. However, there is a heterogeneity in the choice of study variables, which may affect the uniformity of the results.

The findings of this review demonstrate that the Robin Hood Index (RHI) has been used with considerable heterogeneity across studies, both in terms of adjustment variables and inequality outcomes. This inconsistency poses challenges for comparative interpretation and weakens the ability to derive generalizable conclusions about its utility as a standardized measure of health equity. While some studies reported associations between redistribution efforts (e.g., physician supply, income distribution) and inequality outcomes, the overall evidence remains inconclusive and often methodologically fragmented.

Importantly, the pooled estimate from the meta-analysis suggests a non-significant trend toward increased inequality in contexts of resource redistribution. This counterintuitive pattern may reflect complex real-world dynamics, such as implementation lag, reverse causality, or structural barriers in deprived areas. Taken together, these findings underscore the need for greater methodological standardization, clearer theoretical framing, and more robust use of the RHI in future research.

We found that the studies used in our research differ among themselves and can be classified into three categories based on their characteristics. This emerged from the systematic review, but mainly from the study using KNIME, where k-means clustering revealed three categories: the redistribution studies of the number of physicians [3,5,6,9,10,11,12,24,26,27], the systematic review [8], and the poverty–mortality studies [2,4,7,14,23,25]. Studies use RBI in health inequalities but show heterogeneity.

Regarding the percentage distribution of data, we observed that the clustering of studies also affects the extent to which data appear in the results. Regarding the differentiation of the distorted distribution of physicians and the redistribution of physician numbers in relation to income redistribution and the health-poverty nexus, we note that this is due to the heterogeneity of the studies. From the study of the correlation coefficients, we see that the greater the number of variables, the more they are correlated only with some of the key data categories of the study. This probably indicates that these studies [2,4,8,17], which use the largest number of variables, also require a more detailed correlation with the examination of other critical variables (redistribution of the number of doctors, reference to equality/inequality) for health disparities studies. The use of data analysis methods in inequality measurement studies [2,4,11,25,27] is positively correlated with various data categories. It is recommended that future researchers employ a larger number of variables in disparity studies and utilize more sophisticated statistical methods to analyze the field of health disparities more comprehensively in these ways.

Regarding small sample sizes, some studies in our review feature smaller sample sizes compared to others, which may limit the generalizability of their findings. In terms of study availability, the category of systematic reviews contains only one study, reflecting the limited existing research on this topic, which underscores the need for further investigation to strengthen the evidence base.

Studies with RHI may in the future be clearly distinguished by type to facilitate inference for each category. We also consider it important for future researchers to study the correlation between inequalities and policies, the emphasis on marginalized groups, and the persistence of inequality.

The fact that no study reaches the limits of certainty, as it emerges from the data analysis, with the forest plot, makes us cautious about the absolute formulation of the conclusions. However, we believe that the statistical significance shown in the analysis supports the generalization of the results. Additionally, a study that reaches opposite conclusions affects the limits of statistical certainty, and this should be considered.

We examined the effects of missing outcome data from individual participants (due to losses to follow-up or exclusions from analysis) and found no significant results.

### 4.2. Analysis of Adjusted and Non-Adjusted Indicators in the Included Studies

The use of adjusted and no-adjusted indicators in health inequality studies, such as the Robin Hood Index (RHI), is crucial for assessing the validity of results regarding the unequal distribution of doctors. The RHI, adjusted for factors such as age, gender, and socioeconomic conditions, provides more reliable results as it considers factors that affect healthcare needs. In contrast, no-adjusted indicators overlook these differences, leading to potential distortions. In our study, we categorized the studies that used adjusted and non-adjusted indicators and incorporated this categorization into the analysis of results. Adjusted indicators are often used in studies of physician distribution, such as the [5] study, whereas no-adjusted indicators, as seen in the [7] study, do not consider demographic or social factors, thereby underestimating real inequalities. The need for more accurate results, considering social and demographic parameters, led us to primarily use adjusted indicators, as non-adjusted studies could lead to unjustified conclusions.

Our study focused on the gap between adjusted and non-adjusted studies to ensure the accuracy of the meta-analysis results, examining the impact of parameters such as age and gender. Although we tried to use data from studies that provided adjusted data, this was not possible for studies without such characteristics. Therefore, we applied sensitivity analysis techniques to evaluate the impact of studies without adjustment, demonstrating how they affect the results.

The discussion on sensitivity analysis in the meta-analysis study includes commentary on some notable findings. Among all the studies examined, it is confirmed that an increase in the number of doctors significantly increases inequalities, with an overall average odds ratio of 1.24020 (Cl: 0.54–2.86). However, applying the criterion for sensitivity analysis strengthens this finding, modifying the overall odds ratio to 1.26240 (Cl: 0.56–2.89). The application of strict criteria regarding the inflexible use of adjusted indicators overturns this finding, showing a reduction in inequalities with the increase in the number of doctors and setting the overall odds ratio at 0.72306 (Cl: 0.38–1.38). However, a limitation appears due to the reduction in the number of studies in the advanced levels of sensitivity analysis. Further research is required in the future, which will include additional studies on the impact of increasing the number of doctors on the effects of health inequality. The observed heterogeneity (I^2^ = 67.2%) reflects underlying differences in study design, population characteristics, and measurement of the Robin Hood Index. This level of heterogeneity is expected, given the methodological diversity in the literature, and highlights the need for harmonized approaches to measuring inequality.

Furthermore, the removal of non-adjusted studies reduced the heterogeneity between studies and enhanced the reliability of the results, allowing for the creation of more consistent conclusions. This improved the validity of the results, as adjusted indicators provide a more realistic and representative picture of inequalities, avoiding distortions that arise from using non-adjusted indicators.

Our research leaves room for future improvement, primarily through the inclusion of more refined studies to better address the social and demographic variables that influence the distribution of doctors. Expanding research in this direction will enable the creation of more reliable and accurate results on healthcare inequalities.

### 4.3. Limitations, Application, and Future Research

#### 4.3.1. Limitations

From the literature review of the studies, we found considerable methodological heterogeneity in study design, outcome measurement tools, and risk of bias. This methodological heterogeneity, or methodological diversity, which makes the research difficult, was addressed through discussion to extract data relevant to the research question of our study.

#### 4.3.2. Suggestions for Future Research Activity in the Field

Future research should carefully examine the variables reported in this study. A larger number of the most frequently used variables should be employed. Emphasis should also be given to less frequently used variables. Also, the mortality poverty study and physician redistribution branches of the RBI studies on health inequalities should be distinguished.

#### 4.3.3. Practical Application of the Results

The results of this study can be applied in practice by political decision-makers to inform decisions about the redistribution of resources, particularly for physicians, in times when the number of doctors is increasing. In this way, the phenomenon of over-accumulation of physicians around large metropolitan centers and the widening of health inequalities can be prevented.

#### 4.3.4. Applying the Results of Our Study to a Broader Context

The framework of health inequalities can be expanded by incorporating the results of our research. This can be carried out primarily by incorporating results regarding the effect of physician clustering around metropolitan centers.

#### 4.3.5. Need for Further Studies to Strengthen the Result

More studies that emphasize the effect of physician growth on health disparities should be conducted. Studies should emphasize, from their design stage, the collection of reliable and more complete data on the increase in the number of multi-specialty physicians to draw more extensive conclusions.

### 4.4. Future Directions for Research

Given the methodological heterogeneity identified in the included studies, future research should aim to standardize the use of the Robin Hood Index (RHI) in measuring health inequalities. Comparative studies across different healthcare systems (e.g., universal vs. market-driven models) would provide deeper insights into structural determinants of inequality. Moreover, integrating RHI with multidimensional indices—such as the Gini coefficient, accessibility measures, and social deprivation indices—may enhance its interpretive power. Ultimately, prospective studies utilizing individual-level data could provide support for causal inferences regarding the impact of physician distribution on population health outcomes.

## 5. Conclusions

This research found that the research question was answered negatively: Increasing the number of doctors does not result in a reduction in health inequalities, but in an increase. This association shows statistical significance, but not statistical certainty. Additionally, a negative answer to the secondary research question was found: studies on inequality with RHI do not yield homogeneous measures to address inequality. Methodological heterogeneity and the classification of studies that calculate health inequality using RHI were established in three categories according to their characteristics. The reasons are the concentration of doctors around large metropolitan and urban centers. Some elements used in RHI studies require further development and disaggregation. Based on the correlation between the data, there is a need to generalize certain categories of data and to expand the use of data analysis methods in future research. The application of strict criteria regarding the use of adjusted indicators in studies significantly limits the ability to draw safe and definitive conclusions that apply to all cases without exception. The contribution of our research to policymakers and its societal impact is significant. Additional research is needed to develop integrated health efficiency frameworks with measurements in the future.

## Figures and Tables

**Table 1:** Overview of empirical studies examining the relationship between income inequality and health outcomes.

1st Author	Years	Study Population	Inequality Measure	Health Outcomes	Adjustment for + Physicians
Kennedy (1996) [4]	1990	250,100,000	RBI-Gini	Mortality Smoking rates Mortality	Population Income household size
Kawachi (1997) [2]	1990	281,421,906	RBI-Gini	Mortality Smoking rates Fertility	Income household size poverty
Gravelle (1998) [11]	1974– 1995	45,919,456	RBI	(Only physicians)	Population Physicians, doctors, or GPs
Szwarcwald (1999) [14]	1996	6,094,183	RBI-Gini	Mortality Neonatal deaths Fertility	Population Income Poverty
Wilkinson (2000) [5]	1998	18,588,580	RBI	Mortality	Population Physicians, doctors, or GPs
Johnston (2001) [24]	1986–1996	17,877,891	RBI	Mortality	Population Physicians, doctors, or GPs per capita physicians
Shi (2003) [25]	1980–1995	281,421,906	RBI-Gini	Mortality	Population household size poverty
Lynch (2004) [8]	1989–1991 1988–1993	281,421,906	RBI-Gini	Mortality Homicide (violent)	Poverty Income Male LE
Dlouhý (2005) [12]	1990–2002	10,873,553	RBI-Gini	-	Population Physicians, doctors, or GPs Beds
Theodorakis (2006) [9]	2000–2004	3,267,000	RBI-Gini	Mortality	Population Physicians, doctors, or GPs
Omrani-Khoo (2013) [26]	2011	18,585	RBI- Gini	Decile ratio	Population Physicians, doctors, or GPs Beds
Mobaraki (2013) [3]	2005–2009	72,456,140	RBI-Gini	Mortality	Population Income Physicians, doctors, or GPs
Beck (2013) [27]	2010	167,653	RBI-Gini	-	Population Income Household size
Park (2015) [23]	2010–2012	172,398	RBI-Gini	Mortality Decile ratio Smoking rates	Population Income Household size
Rezaei (2015) [10]	2001–2011	75,149,669	RBI-Gini	Decile ratio	Population Physicians, doctors, or GPs beds
Honarmand (2017) [6]	2010–2012	-	RBI-Gini	Mortality Infant mortality	Population Income Physicians, doctors, or GPs
Dlouhý (2018) [7]	2022	10,542,942	RBI-Gini	Decile ratio	Population Physicians, doctors, or GPs Beds

## Data Availability

Our study is a systematic review and meta-analysis that has used data from open-access journals indexed in PubMed or Scopus; therefore, there is no issue of non–open access to the data. The data is freely available to anyone wishing to explore the sources we used in our study, which were extracted using a specialized tool developed based on the JBI Evidence Synthesis tool.

## Supplementary Materials

The following supporting information can be downloaded at: https://www.mdpi.com/article/10.3390/epidemiologia6030035/s1, Supplementary File S1: Search Strategy.

## Abbreviations

The following abbreviations are used in this manuscript:

RHI	Robin Hood Index
