# Supplementary material for: Measuring Health Inequalities Using the Robin Hood Index: A Systematic Review with Meta-Analysis
Source: Epidemiologia (Basel). 2025 Jul 10;6(3):35. doi: 10.3390/epidemiologia6030035 (PMC12285989; doi:10.3390/epidemiologia6030035)
Supplement: Supplementary file 1 [file epidemiologia-06-00035-s001.zip › epidemiologia-3655655-supplementary.docx]

Systematic Review Supplementary File 1: Search Strategy

Measuring Health Inequalities Using the Robin Hood Index:
A Systematic Review with Meta-Analysis

Georgios Farantos *, Athanasios Pitis, Maria Diamantopoulou and Fotini Tzavella

Department of Nursing, School of Health Sciences, University of the Peloponnese, 22100 Tripoli, Greece; a.pitis@go.uop.gr (A.P.); m.diamantopoulou@go.uop.gr (M.D.); tzavella@uop.gr (F.T.)

***** Correspondence: gfarantos@go.uop.gr; Tel.: +30-698-002-3616

## Supplementary File 1: Search Strategy

**Title of Study:**
Measuring health inequalities using the Robin Hood Index: a systematic review with meta-analysis

**Authors:**
Georgios Farantos, Athanasios Pitis, Maria Diamantopoulou, Fotini Tzavella

**Objective of the Search:**
To identify all peer-reviewed studies that utilized the Robin Hood Index (RHI) to assess health inequalities, particularly those examining physician distribution or resource reallocation.

**Databases Searched:**

- PubMed (MEDLINE)
- Scopus
- Web of Science Core Collection
- Google Scholar (limited to top 200 results, screened manually)

**Date of Final Search Execution:**
April 14, 2025

**Search Timeframe:**
From January 1, 2000 to April 14, 2025

**Language Restrictions:**
English only

**Population Filters:**
Human studies only

**Search Strings (as applied in each database):**

**1. PubMed:**
("Robin Hood Index"[Title/Abstract] OR "Pietra Index"[Title/Abstract]) AND ("health inequality"[Title/Abstract] OR "health disparity"[Title/Abstract]) AND ("physician"[Title/Abstract] OR "resource allocation"[Title/Abstract] OR "general practitioner"[Title/Abstract])

Filters: Humans, English, 2000–2025

**2. Scopus:**
TITLE-ABS-KEY("Robin Hood Index" OR "Pietra Index") AND TITLE-ABS-KEY("health inequality" OR "health disparity") AND TITLE-ABS-KEY("physician" OR "resource allocation" OR "general practitioner")

Filters: Article type (Research Article), Language (English), Year (≥2000)

**3. Web of Science:**
TS=("Robin Hood Index" OR "Pietra Index") AND TS=("health inequality" OR "health disparity") AND TS=("physician" OR "resource allocation" OR "general practitioner")

Filters: Language (English), Year Published: 2000–2025, Document Types: Articles

**4. Google Scholar:**
"Robin Hood Index" AND "health inequality" AND "physician" (first 200 results screened manually)

**Deduplication Process:**
All records were exported into a reference management software (Zotero), where duplicate entries across databases were identified and removed automatically and manually.

**Additional Screening Notes:**
Reference lists of key review articles and highly cited studies were hand-searched for eligible additional studies.

**Screening and Selection Protocol:**
All retrieved titles and abstracts were independently screened by two reviewers (GF, AP). Full texts of potentially relevant articles were assessed for eligibility based on predefined inclusion and exclusion criteria described in the main Methods section of the manuscript.

**Software Used:**
Zotero (for reference management), Microsoft Excel (for data charting), KNIME Analytics Platform (for meta-analysis)

This search strategy followed PRISMA 2020 guidelines and is compliant with methodological standards for systematic reviews as outlined by the JBI Manual for Evidence Synthesis.
